# The influence of postharvest UV-C treatment on anthocyanin biosynthesis in fresh-cut red cabbage

**DOI:** 10.1038/s41598-017-04778-3

**Published:** 2017-07-12

**Authors:** Jie Wu, Wen Liu, Li Yuan, Wen-Qiang Guan, Charles S. Brennan, Yang-Yong Zhang, Jie Zhang, Zhi-Dong Wang

**Affiliations:** 1 Key Laboratory of Agro-products Processing, Institute of Food Science and Technology, Ministry of Agriculture, Chinese Academy of Agricultural Sciences, Beijing, 100193 China; 2 College of Life Science, Lin Yi University, Linyi, 276000 China; 3 Tianjin Key Laboratory of Food Biotechnology and Food Sciences, Tianjin University of Commerce, Tianjin, 300134 China; 4 Centre for Food Research and Innovation, Department of Wine, Food and Molecular Biosciences, Lincoln University, Lincoln, 85084 New Zealand; 5 Institute of Vegetables and Flowers, Chinese Academy of Agricultural Sciences, Beijing, 100193 China

## Abstract

Red cabbage (*Brassica oleracea* L. var. *capitata* f. *rubra* DC.) is a fresh edible vegetable consumed globally that contains high levels of antioxidant compounds including anthocyanins. In this study, fresh-cut red cabbage was treated with different Ultraviolet-C (UV-C) dosages. Fifteen cyanidin derivatives were observed in UV-C treated fresh-cut red cabbage; four of these were anthocyanins absent in control samples. The optimum dose of UV-C for enhancing total anthocyanin content in fresh-cut red cabbage was 3.0 kJ/m^2^. Different UV-C irradiation doses resulted in miscellaneous responses for each of the anthocyanin compounds, and these alterations appeared to be dose-dependent. The expression of genes relating to anthocyanin metabolism was altered by UV-C irradiation. For example, genes for biosynthetic enzymes including glycosyltransferase and acyltransferase, as well as R2R3 MYB transcription factors (*production of anthocyanin pigment 1* and *MYB114*), had strongly increased expression following UV-C treatment. These results are in accord with the roles of these gene products in anthocyanin metabolism. This is, to the authors’ knowledge, the first report demonstrating that UV-C treatment can increase the antioxidant activity in fresh-cut red cabbage in storage. Moreover, our detailed phytochemical and gene expression analysis establish specific roles for both anthocyanins and metabolism genes in this process.

## Introduction

Ultraviolet C (UV-C) is commonly used for sterilization to maintain the quality and safety of fresh and fresh-cut produce in food supply chains^1^. Recent studies conducted with UV-C as a postharvest treatment in fruits and vegetables have reported that UV-C reduced decay, delayed senescence, increased antioxidant activity, and induced the biosynthesis of several secondary metabolites, such as flavonoids^2–8^. Therefore, the focus of research has recently shifted from the safety of sterilization towards potential improvements in food quality. Studies have indicated that UV-C treatment at 0.43–6.45 kJ/m^2^ increased the content of flavonols and anthocyanins in blueberries^9^, and treatment at 4.1 kJ/m^2^ induced the accumulation of anthocyanins in strawberries^10^. UV-C irradiation was also reported to positively enhance the accumulation of flavonols in grape berry skin^11^. The increases in flavonoid accumulation induced by UV-C irradiation are also known to contribute to increased antioxidant activities and radical scavenging properties in fruits and vegetables^9, 10, 12^.

In recent years, customers have been increasingly demanding fresh-cut or minimally processed fruits and vegetables^13^. Red cabbage (*Brassica oleracea* L. var. *capitata* f. *rubra* DC.) is a popular fresh-cut vegetable with high nutritional value relating to the antioxidant and anti-inflammatory activity of anthocyanins and other plant natural products^2^. It exhibits purple-red colors owing to its accumulation of abundant cyanidin-based anthocyanins; these are acylated by one or two acids from among cumaric acid, sinapic acid, and caffeic acid^2, 14, 15^. High levels of these biologically-active components impart various health promotion and disease prevention qualities^15, 16^. Red cabbage is also widely used in food processing to increase the aesthetic value of food as a natural colorant and to offer health benefits via its dietary antioxidants^17, 18^. The increased demands for fresh-cut vegetables, together with the potential beneficial health effects of red cabbage, have provided new opportunities for the use of fresh-cut red cabbage.

Despite the numerous studies of the effects of UV-C treatment on natural antioxidants in fruits and vegetables, information is scant on the effects on fresh-cut red cabbage; the molecular mechanisms through which UV-C may affect the biosynthesis of anthocyanins in red cabbage remain elusive. It is now firmly established that the plant anthocyanin biosynthesis pathway starts with chalcone synthase (CHS), followed by the reactions catalyzed by chalcone isomerase (CHI), flavanone 3-hydroxylase (F3H), flavonoid 3′-hydroxylase (F3′H), dihydroflavonol 4-reductase (DFR), and anthocyanidin synthase (ANS). Once the cyanidin-based anthocyanins are formed, they can be further modified by specific glucosyltransferase (GT) and acyltransferase (AT) in a species-specific manner to produce various highly acylated anthocyanins^19–21^. Anthocyanin metabolism is under the complex regulation of diverse regulatory genes at the transcriptional level^20–22^. However, the transcriptional-level responses of genes in anthocyanin metabolism to UV-C treatment remain unexplored.

Most researchers and purveyors both recommend consuming fresh-cut produce within 7 days^23–25^. A maximum storage time of between 7 and 12 days was also reported for fresh-cut vegetables under applicable storage conditions^26–31^. However, there is a dearth of information in the scientific literature regarding storage time for fresh-cut cabbage. We thus used the reported storage times of other fresh-cut vegetables such as celery^28^ and lettuce^31^ to guide our experimental design. The present study sought to evaluate not only the changes in the total anthocyanin content and in the total antioxidant activity in UV-C treated fresh-cut red cabbage, but also set out to examine the responses of individual anthocyanin compounds and particular genes of the anthocyanin biosynthesis pathway to various doses of UV-C treatment during prolonged storage (up to 12 days). Our results suggest that the observed responses of fresh-cut red cabbage to UV-C can potentially be attributed to the differential expression of biosynthetic enzymes and MYB transcription factors and their effects on the accumulation of acylated anthocyanins present in fresh-cut red cabbage.

## Results

### Identification of anthocyanins in fresh-cut red cabbage

Fresh-cut cabbage treated with a predetermined dose of UV-C (0, 1.0, 3.0, or 5.0 kJ/m^2^) were stored for 1, 4, 8, and 12 days. To examine the composition and content of anthocyanins in fresh-cut red cabbage during storage following UV-C treatment, HPLC analysis was performed and each compound was identified via comparison with previously-published data according to chromatographic behavior, UV-Vis spectra, and MS fragmentation patterns.

**Figure 1:**
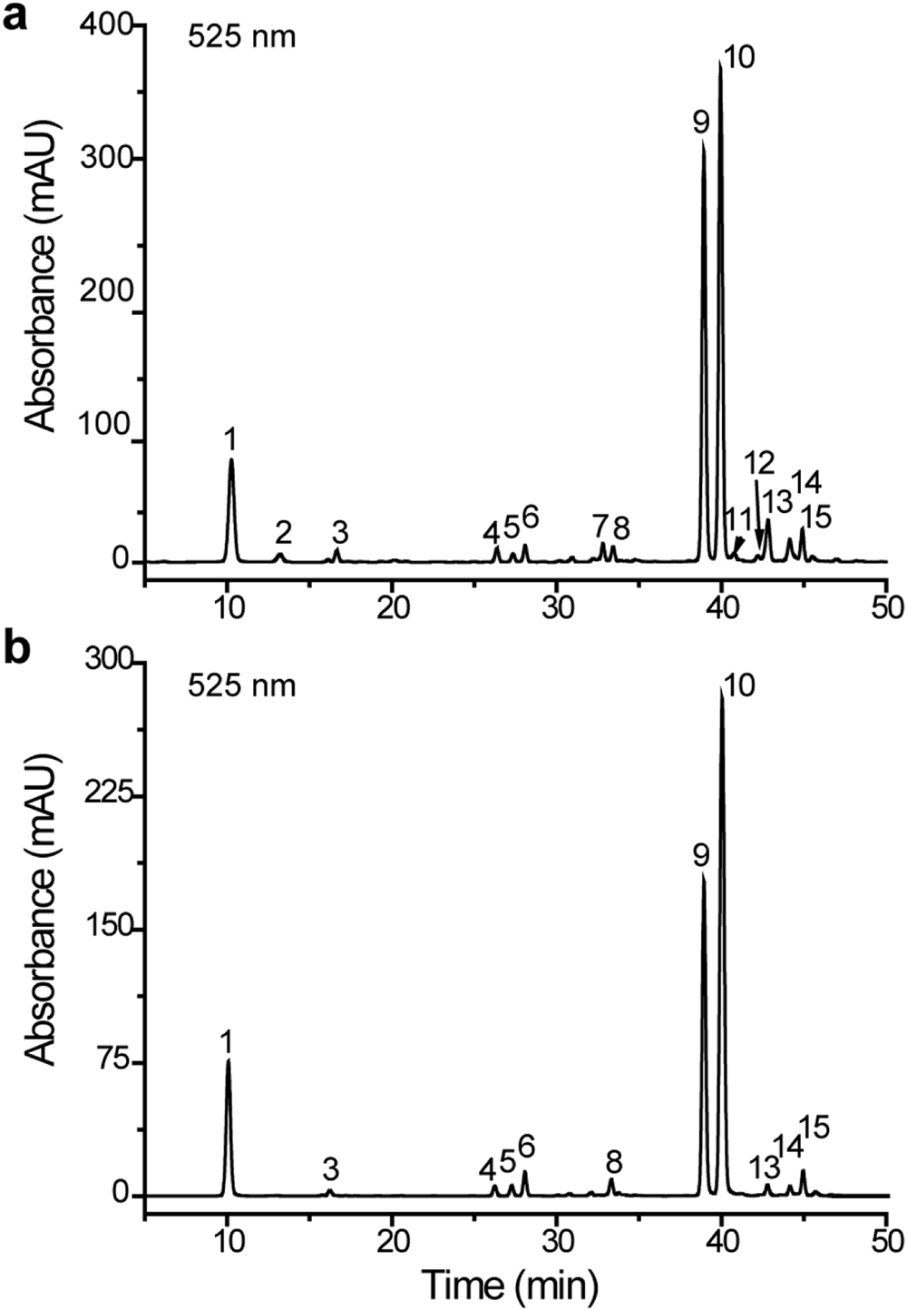
HPLC chromatograms of anthocyanins in fresh-cut red cabbage detected at 520 nm. (**a**) The HPLC profiles of anthocyanins from UV-C treated (3.0 kJ/m^2^) fresh-cut red cabbage after 8 days of storage. (**b**) The HPLC profiles of anthocyanins in untreated fresh-cut red cabbage. Peak assignments are listed in Table 1.

The HPLC chromatograms of the UV-C treated red cabbage were characterized by three major peaks and 12 minor peaks (Fig. 1a). As shown in Table 1, all fifteen compounds had fragment ions at *m/z* 287 and E_440_/E_vis-max_ values ranging from 17% to 19%, demonstrating that these were cyanidin-3,5-*O*-glycosides^32, 33^. The characteristic shoulder in the UV spectra at 314 to 334 nm identified peaks 3–15 as acylated anthocyanins with aromatic acids^34^. Accordingly, the three major peaks (peak 1, 9, and 10) were identified as Cy-3-*O*-diglucoside-5-*O-*glucoside (Cy3diG5G), Cy-3-*O*-(*p*-coumaroyl)-diglucoside-5-*O*-glucoside (Cy3*p*CdiG5G), and Cy-3-*O*-(sinapoyl)-diglucoside-5-*O*-glucoside (Cy3(si)diG5G), respectively (Table 1)^15, 33^. The other minor peaks detected in UV-C treated fresh-cut red cabbage samples were also identified as different forms of cyanidin anthocyanins, based on the information in Table 1 and via comparison with previous reports^15, 35^.

**Table 1:** The UV-Vis and MS data of the anthocyanin compounds identified from red cabbage in this study.

Peak	t_R_ (min)^a^	λ_vis_ (nm)	λ_acyl_ (nm)	E_440_/E_vis_ (%)^b^	E_acyl_/E_vis_ (%)^c^	[M]^+^ (*m/z*)	Fragment ion (*m/z*)	Tentative identification	Abbreviation
1	10.265	514	—	18	—	773	611/449/287	Cy-3-*O*-diglucoside-5-*O*-glucoside	Cy3diG5G
2	13.218	515	—	18	—	611	449/287	Cy-3-*O*-glucoside-5-*O*-glucoside	Cy3G5G
3	16.653	528	334	18	66	979	817/449/287	Cy-3-*O*-(sinapoyl)-diglucoside-5-*O*-glucoside	Cy3(si)diG5G
4	26.373	524	—	17	—	1081	919/449/287	Cy-3-*O*-(caffeoyl)-(*p*-coumaroyl)-diglucoside-5-*O*-glucoside	Cy3(ca)*p*CdiG5G
5	27.350	524	324	17	67	1111	949/449/287	Cy-3-*O*-(feruloyl)-triglucoside-5-*O*-glucoside	Cy3(fe)triG5G
6	28.086	524	332	17	46	1141	979/449/287	Cy-3-*O*-(sinapoyl)-triglucoside-5-*O*-glucoside	Cy3(si)triG5G
7	32.809	522	332	18	40	919	757/449/287	Cy-3-*O*-(*p*-coumaroyl)-diglucoside-5-*O*-glucoside	Cy3*p*CdiG5G
8	33.424	522	332	18	45	935	773/449/287	Cy-3-*O*-(caffeoyl)-diglucoside-5-*O*-glucoside	Cy3(ca)diG5G
9	38.932	522	314	17	60	919	757/449/287	Cy-3-*O*-(*p*-coumaroyl)-diglucoside-5-*O*-glucoside	Cy3*p*CdiG5G
10	39.945	522	330	18	53	979	817/449/287	Cy-3-*O*-(sinapoyl)-diglucoside-5-*O*-glucoside	Cy3(si)diG5G
11	40.758	522	308	17	64	787	287/449	Cy-3-*O*-(feruloyl)-glucoside-5-*O*-glucoside	Cy3(fe)G5G
12	42.208	522	324	17	55	817	449/287	Cy-3-*O*-(sinapoyl)-glucoside-5-*O*-glucoside	Cy3(si)G5G
13	42.829	532	322	19	94	1125	963/449/287	Cy-3-*O*-(feruloyl)(feruloyl)-diglucoside-5-*O*-glucoside	Cy3(fe)(fe)diG5G
14	44.143	530	320	18	91	1155	993/449/287	Cy-3-*O*-(feruloyl)(sinapoyl)-diglucoside-5-*O*-glucoside	Cy3(fe)(si)diG5G
15	44.903	534	332	18	94	1185	1023/449/287	Cy-3-*O*-(sinapoyl)(sinapoyl)-diglucoside-5-*O*-glucoside	Cy3(si)(si)diG5G

^a^t_R_, retention time.

^b^E_440_/E_vis_, the ratio of the absorbance value at 440 nm and that of the maximum visible absorption wavelength.

^c^E_acyl_/E_vis_, the ratio of the absorbance value at the corresponding maxima.

Interestingly, peaks 3 and 10 and peaks 7 and 9 (Table 1), have very similar absorption spectra and patterns of MS/MS fragmentation, suggesting that these may be pairs of isomers that differ in the positions of acylation and or the type of sugar-sugar linkage, which would result in differential retention time^36, 37^. Therefore, both compounds from each of these pairs were assigned as Cy3(si)diG5G and Cy3*p*CdiG5G, respectively.

### Changes in the anthocyanin composition in UV-C treated fresh-cut red cabbage

**Figure 2:**
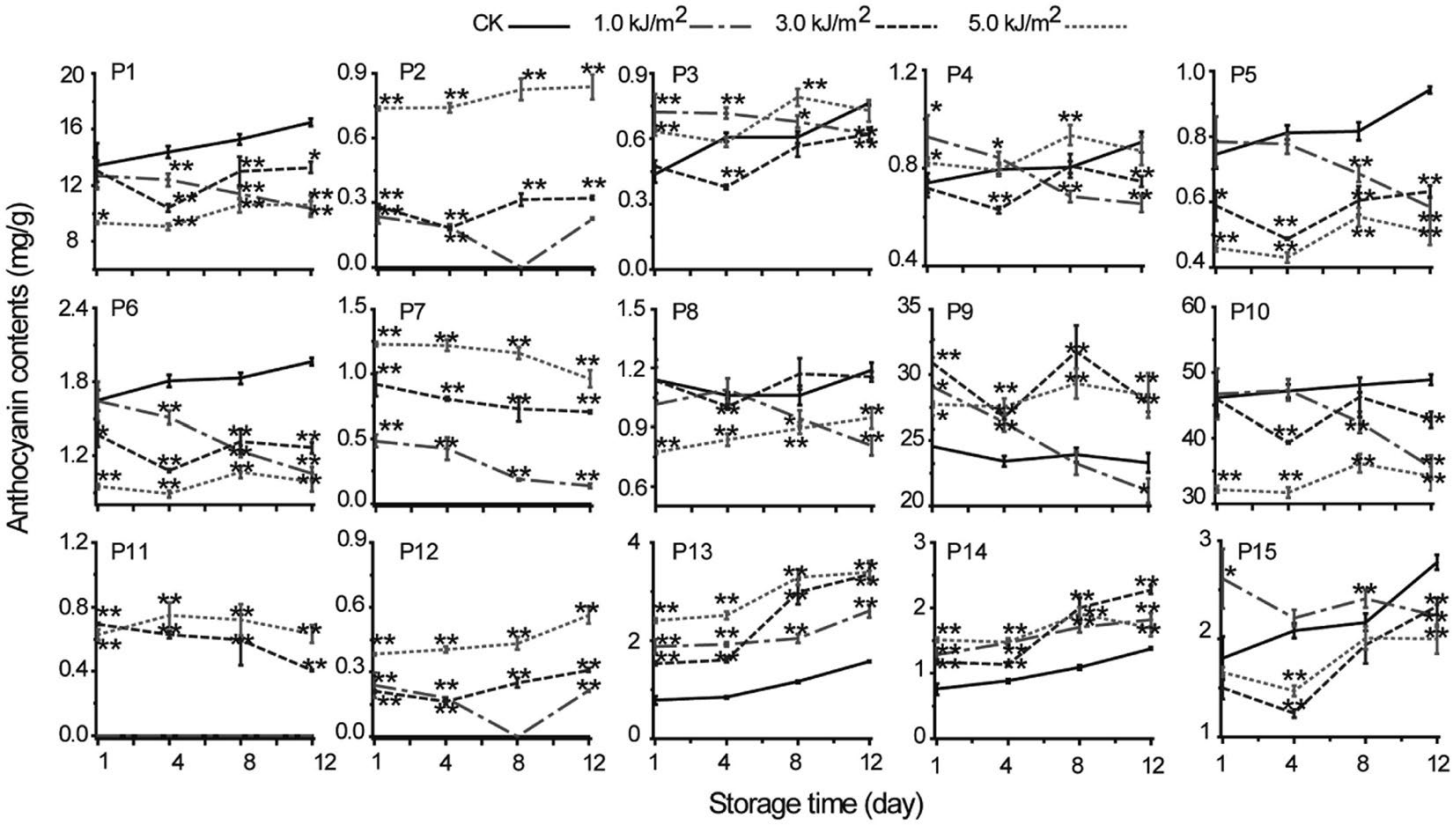
The accumulation of each anthocyanin compound assessed during the storage period following different UV-C treatment doses. The content (mg/g dry weight) of each anthocyanin (P1–P15, representing peaks 1–15, respectively) during the period of storage (from 1 to 12 days) following different UV-C doses (0, 1.0, 3.0, 5.0 kJ/m^2^). Compound identification assignments are presented in Table 1. The graph shows the average values of three independent experiments with error bars indicative of the standard deviation (SD). *P < 0.05; **P < 0.01.

The untreated control samples were observed to accumulate 11 anthocyanin compounds (Fig. 1b), while 15 anthocyanin compounds were indentified from UV-C treated fresh-cut red cabbage samples (Fig. 1a). In untreated samples, Cy3(si)diG5G (peak 10) was the major compound (50.02% of the total anthocyanin content) followed by Cy3*p*CdiG5G (peak 9, 26.63%), Cy3diG5G (peak 1, 14.60%) and several minor peaks (Supplementary Table S1). These cyanidin derivatives were also the main components in UV-C treated fresh-cut red cabbage at different time intervals during storage. However, several new, minor peaks of cyanidin derivatives (peaks 2, 7, 11, and 12) were also recorded exclusively in the UV-C treated samples (Fig. 1a). These profiles with 15 cyanidin derivatives were observed for almost all of the UV-C treated samples during storage, with the exception of peak 11, which was absent in samples processed with low doses of UV-C (1.0 kJ/m^2^) (Fig. 2). These results clearly indicated that UV-C treatment affects the phytochemical composition of fresh-cut red cabbage.

### Changes in the content of each anthocyanin in UV-C treated red cabbage during storage

The effect of UV-C treatment on the individual cyanidin derivatives present in red cabbage was studied in detail here (Fig. 2). The content of individual compounds differed depending on the different UV-C doses applied. The accumulation of P2 (Cy-3-*O*-glucoside-5-*O-*glucoside), P11 (Cy-3-*O*-(feruloyl)-glucoside-5-*O*-glucoside), and P12 (Cy-3-*O*-(sinapoyl)-glucoside-5-*O*-glucoside) increased as the intensity of the UV-C dose increased. On the contrary, higher UV-C radiation doses resulted in decreased accumulation of P1 (Cy3diG5G), P5 (Cy-3-*O*-(feruloyl)-triglucoside-5-*O*-glucoside), P6 (Cy-3-*O*-(sinapoyl)-triglucoside-5-*O*-glucoside), and P10 (Cy3(si)diG5G) during storage. When the UV-C dose reached 5.0 kJ/m^2^, the content of the mentioned four compounds (P1, P5, P6, and P10) decreased dramatically. This suggests that the presence of the anthocyanins with a lower degree of glucosylation or acylation (P2, P11, and P12) may result from the UV-C treatment-induced photodegradation of anthocyanins with a higher degree of glucosylation or acylation (P1, P5, and P6 or P10).

Treatment with UV-C significantly increased the amount of compounds P7 (Cy3*p*CdiG5G), P9 (Cy3*p*CdiG5G), P13 (Cy-3-*O*-(feruloyl)(feruloyl)-diglucoside-5-*O*-glucoside), and P14 (Cy-3-*O*-(feruloyl)(sinapoyl)-diglucoside-5-*O*-glucoside). Changes in the levels of these compounds in responses to UV-C treatment during storage appeared to be dose-dependent. However, when treated with 1.0 kJ/m^2^ UV-C, the levels of compounds P7 and P9 decreased markedly during storage. This result agrees with previous research indicating that monoacylated compounds are less stable than diacylated anthocyanins during storage in red cabbage^17^. The intensity of the radiation doses of UV-C appeared to be directly related to the accumulation levels of most of the anthocyanin derivatives in fresh-cut red cabbage. Moreover, these results demonstrated here shows that higher UV-C doses correlates well with the higher accumulation of the four anthocyanins (P7, P9, P13, and P14) observed throughout the storage period. However, when treated with 3.0 kJ/m^2^ UV-C, some compounds (for example, P6, P8–P10, and P14–P15) exhibited higher levels of accumulation than those when treated with either 1.0 or 5.0 kJ/m^2^ UV-C after 8 days of storage. Thus, results indicate that treatment with intermediate irradiance levels of 3.0 kJ/m^2^ seems to be the optimum UV-C level that can contribute to the accumulation of anthocyanins in fresh-cut red cabbage.

### UV-C treatment increased the total anthocyanin content in fresh-cut red cabbage

The total anthocyanin content in untreated fresh-cut red cabbage ‘*ZiGuang*’ was 92.96 ± 2.26 mg/g DW (Supplementary Table S1), and this value markedly increased following treatment with 1.0 and 3.0 kJ/m^2^ UV-C (Supplementary Fig. S1). However, treatment with 5.0 kJ/m^2^ UV-C did not further enhance the level of total anthocyanins. The total anthocyanin content reduced gradually during the course of storage following treatment with 1.0 kJ/m^2^ UV-C. Treatment with 3.0 kJ/m^2^ UV-C caused the total content of anthocyanins to peak at 8 days of storage (105.42 ± 3.63 mg/g DW, Supplementary Table S1). These results suggest that appropriate doses of UV-C treatment (such as 3.0 kJ/m^2^) can significantly increase the total anthocyanin content in red cabbage, and this treatment may have residual effects that are sustained during the prolonged storage period.

### The effect of UV-C treatment on the antioxidant activity of fresh-cut red cabbage

The total antioxidant capacity (TAC) of the soluble and insoluble fractions of fresh-cut red cabbage samples collected during storage following UV-C treatment were assayed by the global antioxidant response (GAR) method supplied with FRAP assays. Compared to the control, significantly higher TAC values were detected in both the soluble and insoluble fractions of samples treated with 3.0 kJ/m^2^ UV-C after one day of storage (Supplementary Fig. S2). The 1.0 kJ/m^2^ UV-C doses also increased the TAC, but to a lesser extent for this same time period. A decline (not significant) in the TAC value was observed with the 5.0 kJ/m^2^ UV-C dose. Seemingly then, 3.0 kJ/m^2^ UV-C can increase the antioxidant activity of fresh-cut red cabbage. These results are in accordance with the changes observed in the total anthocyanin content following treatment with UV-C. After 12 d of storage, 5.0 kJ/m^2^ UV-C also significantly increased the TAC in the soluble fractions.

The soluble fraction was the main contributor to the TAC in the samples (more than the 80% of the activity), whereas the insoluble fraction accounted for 16–20% of the TAC. However, there were no obvious changes in the TAC values of the insoluble fraction at 4, 8, and 12 days. These results suggest that water-soluble polyphenols, including anthocyanins, are the major contributing compounds to the TAC of red cabbage.

### UV-C treatment influences the expression profiles of anthocyanin biosynthetic and regulatory genes in fresh-cut red cabbage

**Figure 3:**
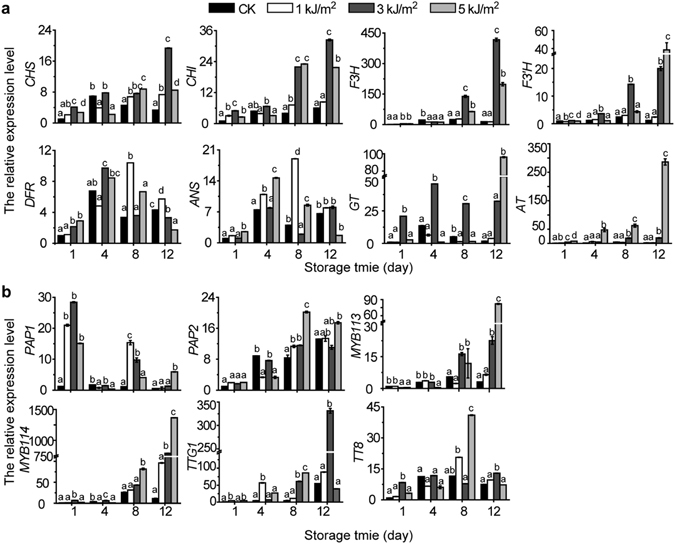
Expression analysis of anthocyanin biosynthetic genes and regulatory genes in UV-C treated fresh-cut red cabbage. The relative expression levels of anthocyanin biosynthetic genes (**a**) and regulatory genes (**b**) in fresh-cut red cabbage treated with UV-C were analyzed at the indicated storage days. The enzyme names encoded by the biosynthetic genes analyzed in this study are as follows: CHS, chalcone synthase; CHI, chalcone isomerase; F3H, flavanone 3-hydroxylase; F3′H, flavanone 3′-hydroxylase; DFR, dihydroflavonol 4-reductase; ANS, anthocyanidin synthase; GT, glucosyltransferase; AT, acyltransferase. The expression of MYB family transcription factors (*PAP1*, *PAP2*, *MYB113*, and *MYB114*) and the transparent testa genes (*TTG1* and *TT8*) are also displayed. Expression values have been normalized with the *tublin* gene. The graph shows average values of three replicates, with error bars representing the standard error of the mean (n = 3) and different letters indicate significance at *P* < 0.05.

To investigate the molecular mechanisms underlying the observed anthocyanin accumulation changes in fresh-cut red cabbage following treatment with UV-C, the expression profiles of several anthocyanin biosynthetic and regulatory genes were examined by qRT-PCR. The expression levels of eight biosynthetic genes (*CHS*, *CHI*, *F3H*, *F3′H*, *DFR*, *ANS*, *GT*, and *AT*) and six regulatory genes (*PAP1*, *PAP2*, *MYB113*, *MYB114*, *TTG1*, and *TT8*) in red cabbage are shown in Fig. 3. In comparison with the untreated fresh-cut red cabbage samples, the expression of most of the anthocyanin biosynthetic and regulatory genes in UV-C treated fresh-cut red cabbage were slightly up-regulated by different doses of UV-C. Among the up-regulated genes on day 1, *GT* exhibited the highest fold increase (20-fold increase relative to the control) by treatment with 3.0 kJ/m^2^ UV-C (Fig. 3a). In the results shown in Fig. 3b, *PAP1* was the only regulatory gene greatly up-regulated by UV-C treatment on day 1, which were about 28-fold higher in samples supplemented with a 3.0 kJ/m^2^ UV-C dose and were 20-fold and 15-fold higher following the 1.0 and 5.0 kJ/m^2^ UV-C doses, respectively, when compared to untreated controls. Thus, the increase of total anthocyanin content in UV-C treated fresh-cut red cabbage by 3.0 kJ/m^2^ doses (Supplementary Fig. S1) seems reasonable, and can likely be attributed to the coordinated transcriptional activation of *PAP1* and *GT*.

The induction of the expression of anthocyanin biosynthesis genes appeared to be maintained for a prolonged period in the UV-C treated fresh-cut cabbage samples (Fig. 3). Most of the genes in the anthocyanin biosynthesis pathway exhibited maximal expression levels at the 12 day time point; exceptions to this trend were *DFR*, *ANS*, and *TT8*, which exhibited variable responses to UV-C treatments. These latter genes had maximal expression at day 4 or 8, depending on the UV-C dosage. UV-C doses at 3.0 kJ/m^2^ treatment significantly increased the expression levels of biosynthesis genes (*CHS*, *CHI*, *F3H*, *F3′H*, and *GT*) and some regulatory genes (e.g. *TTG1* and *MYB113*) in anthocyanin biosynthesis were significantly up-regulated during storage. Similarly, 5.0 kJ/m^2^ UV-C treatment significantly up-regulated the expression of *F3′H*, *GT*, and *MYB113* on day 12 of storage. Furthermore, 5.0 kJ/m^2^ UV-C increased the expression of *F3′H* (38-fold increase), *GT* (56-fold increase), *AT* (130-fold increase), *MYB113* (27-fold increase) and *MYB114* (120-fold increase) were particularly strongly affected by this treatment (Fig. 3a,b).

Interestingly, most of the anthocyanin biosynthesis genes that were induced by UV-C treatment showed the most pronounced increased with the 3.0 kJ/m^2^ UV-C doses. However, the expression levels of *AT*, one of the late genes involved in a terminal modification reaction step of the anthocyanin biosynthesis pathway, was progressively and consistently up-regulated as the UV-C doses were intensified. *MYB114* expression exhibited its maximum level with the 5.0 kJ/m^2^ UV-C dose, and this trend was most apparent at day 12. These results suggest that the expression of both *AT* and *MYB114* in UV-C treated fresh-cut red cabbage may be UV-C dose dependent.

## Discussion

### The photodegradation and photoinduction of anthocyanins by UV-C in fresh-cut red cabbage is both durable and dose-dependent

Red cabbage contains an abundance of bioactive substances like acylated anthocyanins; these compounds have high nutritional benefits and health-promoting functions. This study illustrates, for the first time, that the treatment with UV-C affected the composition and content of anthocyanins in fresh-cut red cabbage.

Previous studies have shown that diacylated anthocyanins can be deacylated to form monoacylated or nonacylated derivatives^36, 38^. It is also known that the hydrolysis of glucosidic bonds in anthocyanins led to the formation of derivatives with less extensive glucosylation^36, 39^. These phenomena may explain our observation of the increased accumulation of nonacylated or monoacylated anthocyanins (like P2, P11, and P12), which may result from the UV-C treatment-induced photodegradation of compounds (P1, P5, and P6 and/or P10). Moreover, under higher intensity UV-C dosages photodegradation increased the levels of the aforementioned compounds. Anthocyanins acylated with sinapic acid are known to be less stable than those acylated with ferulic acids^37^. Thus, P11 was present only following treatment with 3.0 and 5.0 kJ/m^2^ UV-C, but other degradation products (P2, P7, and P12) were observed with the1.0 kJ/m^2^ UV-C dose. The trends observed for the responses to the various doses of UV-C were sustained during the entire period of storage. These results indicated that photodegradation of anthocyanins induced by UV-C in fresh-cut red cabbage were generally dose-dependent and, further, that these effects were durable with storage time.

The variations in the accumulation of the several anthocyanin compounds following treatment with UV-C can possibly be explained by the degradation of anthocyanins with higher degree of glucosylation or acylation. However, the accumulation of P7, P9, P13, and P14 may be related to the transcriptional activation of anthocyanin synthesis genes as triggered by UV-C treatment. This photoinduction effect was also dose-dependent and durable through the entire 12 days of storage.

UV-C irradiation has been investigated in various fruits such as blueberries^12^ and strawberries^10, 40^, and it has been demonstrated that treatment with UV-C induced the accumulation of anthocyanins. However, the fruit responses appears to be transient in that UV-C treatment induced a spike in anthocyanin accumulation straight after induction, with the beneficial effects dissipating over time. A previous report has highlighted that the substantial increases in anthocyanins observed in blueberries exposed to UV-C decreased to the same level as the untreated control berries after 19 h^7^. In strawberry, the effects of anthocyanin accumulation induced by UV-C irradiation were only detected three days after the treatments were administered^10^. During further prolonged storage, UV-C irradiation has even been observed to suppress the accumulation of anthocyanins in strawberries to differing extents^40^. In the present study, the accumulation of anthocyanins induced by UV-C treatment in fresh-cut red cabbage, especially for P7, P9, P13, and P14, were sustained during the 12 days of storage. This observation represents new insight about the accumulation of anthocyanins in vegetables.

### Acyltransferases and glycosyltransferases are involved in the responses of fresh-cut red cabbage to UV-C treatment

Many reports have linked UV-A and UV-B to the accumulation of flavonoids in many plants, and the molecular mechanisms underlying these processes have been well established^41–46^. However, there has to date been little research addressing the potential effects of UV-C irradiation on anthocyanin biosynthesis and accumulation^10^. In this study, UV-C treatment induced the expression of a set of anthocyanin biosynthetic genes. Among these up-regulated genes, *GT* was dramatically up-regulated, by 20-fold with the 3.0 kJ/m^2^ UV-C dose. The elevated expression level of *GT* may explain our observation that the accumulation of the diglucosylated anthocyanins P7, P9, P13, and P14 was increased following UV-C treatment. It is notable that the expression level of *AT* was exclusively observed in a dose-dependent response, that was maintained during the entire storage period. It seems that the expression of *AT* is indispensable for fresh-cut red cabbage when exposed to UV-C. Previous studies have shown that the expression of anthocyanin biosynthetic genes was up-regulated by UV-C, but such effects were transient, with most such changes observed within the first 24 h post-treatment^46–48^. In fresh-cut red cabbage, we found that these effects were longer lasting. These findings indicate that the UV-C dose-dependent expression of *AT*, which endows anthocyanins with acylated modifications, may contribute to the durable responses of anthocyanins observed in fresh-cut red cabbage following UV-C treatment. It should be noted that the extent (magnitude) of the differences in the data for the day 4 and day 8 samples was smaller than the data from day 12. A time course with samples collected at points after 12 days would enable researchers to see the highest level of expression for the 14 genes. Therefore, we suggest that future studies may want to collect data for a time series that includes 1, 6, 12, and 18 day time points. This extended time series could help to characterize the full extent of the induction of gene expression that we observed in the present study.

**Figure 4:**
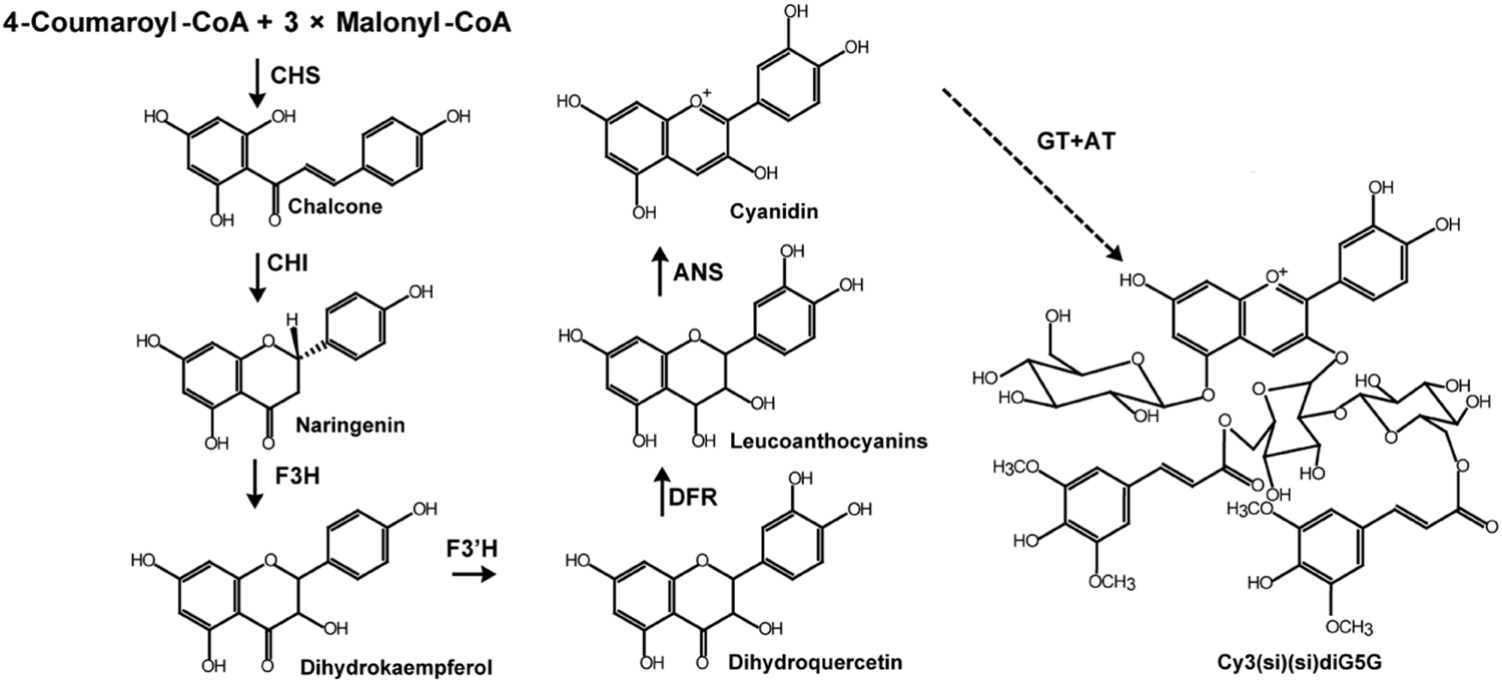
The biosynthetic pathway of the anthocyanins. Scheme displaying the biosynthetic pathway of Cy-3-*O*-(sinapoyl)(sinapoyl)-diglucoside-5-*O*-glucosidein red cabbage. The biosynthetic pathway of anthocyanidins leads to the biosynthesis of cyanidin, which is further modified with several glycosyl and acyl moieties added in a species specific manner by glycosyltransferases and acyltransferases (indicated by dotted lines).

Taking into consideration the previous studies of the anthocyanin biosynthesis pathway^49, 50^ and the compounds detected in the present study, it may be the case that the anthocyanidins in red cabbage undergo modification in a concerted manner, with glycosyl and acyl groups (aromatic acyl groups) putatively catalyzed by glycosyltransferases (GTs) and acyltransferases (ATs), respectively, similar to what is known for other plants^19–21^ (Fig. 4). It is known that glycosylation by GTs enables the subsequent acylation by ATs that ultimately results in the great structural diversity of polyacylated anthocyanins with multiple glycosyl and acyl groups^51^. Considering that the expression of *GT* and *AT* genes are significantly up-regulated over prolonged storage in fresh-cut red cabbage following UV-C treatment, it seems that the specific responses of red cabbage to UV-C are somehow relevant to the polyacylated anthocyanins in red cabbage. Therefore, glycosylation and acylation of anthocyanins by GTs and ATs in fresh-cut red cabbage may play important interactive roles in responses to UV-C treatment. However, the exact functions of particular GTs and ATs in UV-C treated fresh-cut red cabbage will require further confirmation with genetic and biochemical studies.

### Responses to UV-C treatment in fresh-cut red cabbage are probably regulated by transcriptional factors

It has been well established that various types of transcription factors are involved in plant responses to UV-A and UV-B irradiation, with comparatively less known about the effects of UV-C irradiation in plants. In *Arabidopsis thaliana*, the MYB genes *AtPAP1*, *AtPAP2*, *AtMYB113*, and *AtMYB114* are known to directly regulate anthocyanin biosynthesis by activating the transcription of structural genes in a manner coordinated by the bHLH proteins AtTT8 and AtTTG1^52, 53^. In our study of fresh-cut red cabbage, the expression level of *PAP1* was instantly and dramatically up-regulated by UV-C treatment. As reported in *Arabidopsis thaliana*, PAP1 can coordinate with TTG1 to activate anthocyanin biosynthesis by regulating the expression of *PAL* and *CHS*, among other genes^54^; and the coordination between regulatory genes can also regulate the expression of *F3H*, *DFR*, *ANS*, and *GT* in apple^55, 56^. It suggests that in response to UV-C irradiation PAP1 may coordinate with other transcription factors to regulate the expression of anthocyanin synthesis genes, especially for *GT*, in fresh-cut red cabbage. For longer storage time of UV-C treated fresh-cut red cabbage in this study, the magnitude of the changes in the expression of the regulatory genes *MYB114* was the largest and most profound with the 5.0 kJ/m^2^ UV-C dose; this was also observed for the expression pattern of *AT*. These different responses of different transcription factor genes in response to various UV-C treatment intensities suggest that the activities of these regulatory genes may somehow be coordinated with each other to regulate different biosynthetic genes in fresh-cut red cabbage.

Many studies have shown that higher plants utilize multiple sensory photoreceptors to accurately perceive red/far-red light, UV-A/blue, and UV-B light^57–60^. These photoreceptors respond to various forms of light energy and activate various signal transduction cascades to regulate light-dependent responses via transcriptional factors and related gene expression in plants. However, the photoreceptors that may be related to the perception of UV-C energy remain elusive in plants. The responses of different regulatory and biosynthetic genes to UV-C in fresh-cut red cabbage may give hints as to the influences of UV-C irradiation on anthocyanin biosynthesis. This study demonstrated that fresh-cut red cabbage responded to UV-C treatment by elevating the expression of an array of anthocyanin biosynthetic and regulatory genes, which in combination with photodegradation reactions resulted in differential anthocyanin accumulation and changes in antioxidant activities. Thus, it can be concluded that appropriately-selected UV-C treatments can be beneficial and have practical impacts for commercial practices with fresh-cut red cabbage, not only as a germicidal agent to reduce spoilage but also as a quality-accentuating/enhancing method to improve the accumulation of healthy antioxidants such as anthocyanins. These results not only open a new window of opportunity for the use of UV-C to stimulate the accumulation of bioactive compounds, but also enhance our understanding about the effects of UV-C on the anthocyanin biosynthesis and accumulation in fresh-cut red cabbage. Moreover, our extensive phytochemical analysis and our gene expression study together lay the foundation for the mechanistic biochemical and molecular characterization of the post-harvest response of fresh-cut red cabbage to UV-C.

## Methods

### Chemicals

Analytical grade methanol and formic acid were purchased from Beijing Chemical Works (China). Acetonitrile, methanol, and formic acid were purchased in chromatographic grade from Fisher Scientific (USA). Trifluoroacetic acid (TFA; ≥99%) was purchased from Zhanyun Chemical Industry (China). Cyanidin 3,5-*O*-diglucoside was purchased from Extrasynthese (France). For the HPLC analysis, de-ionized water was prepared from a Milli-Q System (Millipore, USA).

### Plant materials

Red cabbage cultivar ‘*ZiGuang*’ (*Brassica oleracea* L. var. *capitata* f. *rubra* DC.) were grown in a green house by a professional vegetable cooperative in the Shunyi District of Beijing. They were planted in the soil between the middle of January to the middle of February. The temperature was not allowed to dip below 25°C. After 115 days of growth, moderately-sized (diameter ranged from 43.5–49.5 cm) mature red cabbages were sampled and were immediately transported to the laboratory after harvest. The wax coating of the red cabbage was peeled, and the root segment was cut off. The edible material was subsequently cut into eight similarly-sized pieces. The six layers of each piece were separated carefully to form individual samples prior to UV-C treatment.

### UV-C irradiation, packaging, and storage

A low-pressure mercury (LPM) UV light reactor was assembled using a UV tube (Ruisente Ltd., China), which delivered UV photons at 253.7 nm. The UV-C dose rate was measured using a digital ILT1700 radiometer (International Light Technologies, USA). Different UV-C illumination doses were obtained by altering the duration of the exposure at a fixed distance. The cabbage layers were placed on a holding tray that was positioned under the UV-C light reactor for 50, 150, or 250 s. The leaves were flipped over so that the various layers received the same dose of radiation. The total radiation doses for each red cabbage side were 1.0, 3.0, and 5.0 kJ/m^2^. After treatment, the fresh-cut cabbage was stored at 4°C in the dark. In this experiment, fresh-cut cabbage that had been treated with UV-C was stored for 1, 4, 8, or 12 days. At day 1, 4, 8, and 12 of storage, the samples were transferred to a freezer at −80°C and freeze dried using a Freeze Dry system (Boyikang, China). Prior to chemical extractions, six leaf layers from each of the 8 cut pieces were combined as a single sample. Each sample combination (control and 3 different UV-C doses, and 4 storage durations) was replicated with three separate cabbages (*n* = 3, total samples in the entire experiment = 48).

### Sample extraction and preparation for chemical analysis

Samples were extracted using a mixture of methanol, water, formic acid, and trifluoroacetic acid (70:27:2:1, v/v/v/v)^61, 62^. They were extracted using a KQ-500DE ultrasonic cleaner (Ultrasonic Instruments, China) at 20°C for 30 min, and were then centrifuged at 8000 rpm for 10 min. The supernatants were combined after three repetitions of these extraction steps. The extracts were evaporated under a nitrogen purge, and the residues were redissolved in methanol. The aqueous solution was subsequently filtered using a 0.22 μm nylon membrane prior to further analysis.

### HPLC analysis of anthocyanins from red cabbage

HPLC-DAD analysis for anthocyanins from red cabbage was performed using an Agilent 1200 with a C_18_ column (250 × 4.6 mm i.d., 5 μm, Shiseido, Japan). The solvent system for the anthocyanin analysis method was modified from a previously-reported method^63^. Eluent A was 10% formic acid in water, and Eluent B was formic acid-acetonitrile-water (10:40:50, v/v/v). The following elution gradient was used: 15% B at 0 min, 15% B at 15 min, 50% B at 45 min, 80% B at 55 min, 15% B at 56 min, and 15% B at 60 min. Ten μL of the sample extract was injected with a constant column temperature maintained at 35°C and a flow rate maintained at 0.8 mL/min. Chromatograms were acquired at 520 nm and photodiode array spectra was recorded from 200 to 800 nm in steps of 2 nm during the HPLC analysis. The compounds in each sample were semiquantitatively measured via linear regression in comparison to a standard curve generated from the analysis of a range of concentrations of a commercial cyanidin 3,5-*O*-diglucoside reference standard. The semiquantitative values for the analyte samples were expressed as milligrams of commercial standard per g of dry weight (DW).

To obtain more information about the anthocyanins present in red cabbage, the UV-C treated and control samples were analyzed via HPLC-MS/MS analysis. The HPLC separation conditions were the same as those described above and used an ACQUITY CSH™ C_18_ column (1.7 µm, 2.1 × 100 mm, Waters, USA). Ten μL of the sample extract was injected, and the flow rate was 0.8 mL/min. HPLC-ESI-MS^n^ analysis were carried out with a Q-TOF Premier instrument. Data for anthocyanins were acquired in positive-ion (PI) mode under the following operating conditions: desolvation temperature: 500°C; desolvation gas (N_2_) rate, 900 L/h; cone gas flow, 50 L/h; cone voltage and capillary voltage were 30 V and 2 kV, respectively. The MS spectra were recorded across an *m/z* range of 100–1200.

### RNA extraction and real time PCR analysis

Samples of the fresh-cut and UV-C treated red cabbage at different days of storage were ground into a powder in liquid nitrogen. Total RNA was extracted from each sample for three biological replicates using Trizol reagent (Takara, Japan). Complementary DNA (cDNA) for qRT-PCR analyses was prepared using a SuperRT cDNA kit (Beijing CoWin Biotech Co., Ltd., China). qRT-PCR was carried out on a LineGene 9600 plus PCR system (Bioer Technology Co., Ltd., China) using UltraSYBR Mixture (Beijing CoWin Biotech). The reactions of the analysis were performed in triplicate, as described previously, with slight modifications^19^. The PCR products of each gene were sequenced, and melt curves of qRT-PCR samples were analyzed to confirm specific amplification. The gene-specific primers used for this qRT-PCR analysis were designed by Primer Premier 5 and are listed in Supplementary Table S2. Their design was based on primers reported in previous studies^19, 64^ and on RNA-Seq results for red cabbage available in NCBI databases. The *tubulin* gene from red cabbage was chosen as a reference gene.

### The global antioxidant response and FRAP analysis

The global antioxidant response (GAR) method by FRAP assays were applied to analyze the total antioxidant capacity (TAC) of the soluble and insoluble fractions of fresh-cut red cabbage samples collected during storage following UV-C treatment. Red cabbage samples (powder) were successively digested by α-amylase, pepsin, pancreatin, and bile salts to stimulate oral, gastric, and intestinal digestion as reported in previous study^65^. After gastrointestinal digestion, soluble (bioaccessible) and insoluble (non-accessible) fractions were separated and were measured by FRAP assay (below). The antioxidant activities of both the soluble and insoluble fractions of samples were both evaluated and combined as the ‘global’ antioxidant response^65^.

The FRAP assay was conducted as described by Benzie and Strain^66^, with slight modifications. The FRAP reagent solution was composed of 10 mM TPTZ solution in 40 mM HCl and 20 mM FeCl_3_. For the soluble fraction, the solution was added to the FRAP reagent and shaken for 30 min at room temperature. While the insoluble fraction-FRAP reagent mixture was shaken for 30 min at 37 °C for the determination of antioxidant activities. The absorbance of the supernatant was measured at 595 nm following centrifugation using a spectrophotometer (ONLAB UV Pro, China). Aqueous solutions of tert-butylhy-droxytoluene (BHT) were used as standard for calibration, and the calibration curve was *Y* (absorbance) = 1.2168 *X* (BHT content) + 0.0129 (*R* ^2^ = 0.9996). The results were expressed as n mol equivalents of BHT per g of sample (nmol BHT g^−1^ DW).

### Statistical analysis

One-way analysis of variance (ANOVA) was performed using SPSS 18.0 (IBM, USA). Additionally, Fisher’s least significant difference (LSD) and Duncan’s tests were also used evaluate the statistical significance of changes observed in the data set, at a significance level of *P* < 0.05. The data are expressed as the mean ± standard error.

## Electronic supplementary material

supplemental data
