# Supplementary material for: The influence of postharvest UV-C treatment on anthocyanin biosynthesis in fresh-cut red cabbage
Source: Sci Rep. 2017 Jul 12;7:5232. doi: 10.1038/s41598-017-04778-3 (PMC5507880; doi:10.1038/s41598-017-04778-3)
Supplement: Supplementary file 1 — supplemental data [file 41598_2017_4778_MOESM1_ESM.pdf]

The influence of postharvest UV-C treatment on anthocyanin biosynthesis in fresh-cut red cabbage

Jie Wu[1], Wen Liu[2], Li Yuan[1], Wen-Qiang Guan[1,3], Charles S. Brennan[4], Yang-Yong Zhang[5], Jie Zhang[1,*], Zhi-Dong Wang[1,*]

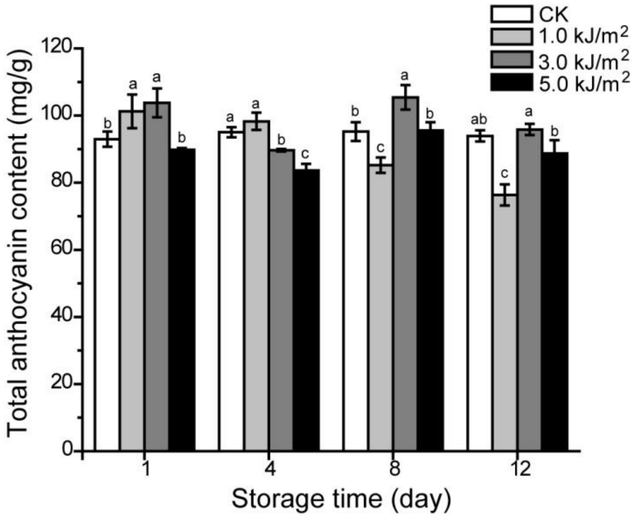

Supplementary Figure S1. Total anthocyanin content during the storage period following various UV-C doses. Plot showing changes in the total anthocyanin content (mg/g dry weight) during the period of storage (from 1 to 12 days) following various UV-C doses UV-C (0, 1.0, 3.0, 5.0 kJ/m^2). The graph shows the mean values ± SD from three independent experiments.

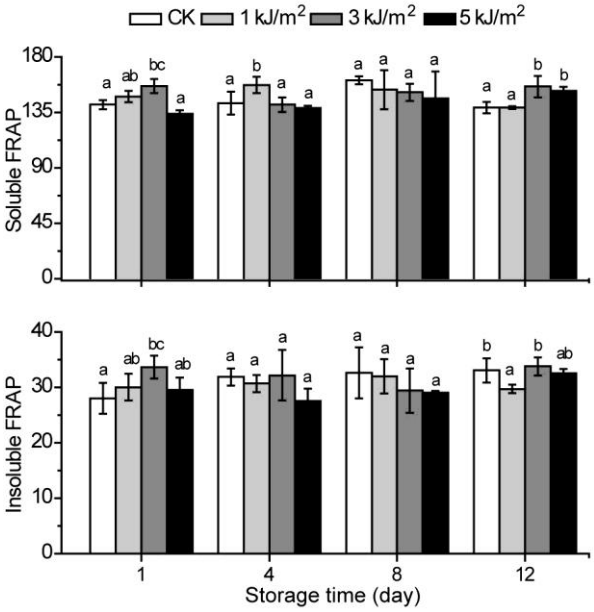

Supplementary Figure S2. Assessment of antioxidant capacity via FRAP assay. The antioxidant capacities of the soluble and insoluble fractions of UV-C treated samples (0, 1.0, 3.0, 5.0 kJ/m^2) in storage (from 1 to 12 days) were analyzed with FRAP assays. The graph shows mean values ± SD of three independent experiments.

Supplementary Table S1. The Content of each anthocyanin in untreated and UV-C treated fresh-cut red cabbage.

Compound[a]	Untreated sample Relative content (%)[b]	UV-C treated sample Relative content (%)[c]
P1	14.60 ± 2.05	12.52 ± 1.42
P2	0	0.30 ± 0.02
P3	0.47 ± 0.03	0.54 ± 0.03
P4	0.80 ± 0.03	0.78 ± 0.02
P5	0.81 ± 0.03	0.58 ± 0.02
P6	1.79 ± 0.07	1.26 ± 0.06
P7	0	0.69 ± 0.07
P8	1.24 ± 0.04	1.12 ± 0.04
P9	26.63 ± 0.79	30.44 ± 0.84
P10	50.02 ± 1.65	44.29 ± 1.35
P11	0	0.58 ± 0.17
P12	0	0.24 ± 0.12
P13	0.85 ± 0.11	2.87 ± 0.34
P14	0.82 ± 0.11	1.93 ± 0.22
P15	1.96 ± 0.28	1.86 ± 0.25
Total content[d]	92.96 ± 2.26	105.42 ± 3.63

[a] Compounds for peaks 1–15 are represented by P1–P15, respectively.

[b] Relative content refers to a percentage of the total anthocyanin content in fresh-cut red cabbage.

[c] Relative content refers to a percentage of the total anthocyanin content in UV-C treated (3.0 kJ/m^2) red cabbage after 8 days of storage.

[d] Values are expressed as milligrams of commercial reference standards per 1 g of dry weight (DW). Data are expressed as means ± SD (n = 3).

Supplementary Table S2. Primers used for the qRT-PCR analysis in this study

Gene	Forward Primer	Reverse Primer
PAL	5′-AGTGTAGGTCGTATCCTTTGTAT	5′-CGCCGTGAAAACCTTATC
C4H	5′-AGAATCCAGACTCTGCGACCAA	5′-CTCTGAGCCAACCTGCTTCTCT
CHS	5′-GGTGGTCCAGCGATCCTTGA	5′-ACTTCCTCCTCATCTCGTCCAA
CHI	5′-ATGATAGCATTCCTGAAACGG	5′-GCAACACTCAACCTAGCCCC
F3H	5′-ATGCCACCACACTCGGACTT	5′- CCTTCAATATACGGCACACGGA
*F3′*H	5′-TTCCGTACCTTCAGGCGGTTATCAA	5′-CTTTGGGGATATGATAGCCGTTGATC
DFR	5′-GCCGCCTAGCCTTATTACCG	5′- AGAATCGTTGCATCGTGAGAGG
ANS	5′-GGTTGAACTAATGGAGCGTGTG	5′-ACTTAGCGTACTCACTCGTAGC
UFGT	5′-GGAGACCACAGGAAGCGATC	5′-CGAGCCAGAAGAACGCATCC
AT	5′-CCTTCCACTCCGCTATCCT	5′-GAAAGTGTGCGAGGACGAG
PAP1	5′- TTCTACCACACCCGCCCAAA	5′-GCATGGAGGAACAACGTCAACT
PAP2	5′-TTCCTTGCTCTTATACCACACC	5′-GTCAGCTTCTGCCATGCCATTA
MYB113	5′-GTCCAAACGGTTGAGAAAAGGTGCAT	5′-CCAGCTCTTAAAGGAACTTGGTGCCAT
MYB114	5′-TGGTGAAGTTGATCTTCTGCTC	5′-CCTGGTTCATGTTTCTTACTCA
TTG1	5′- GAGCACTCCACCATCATCTACG	5′-CCAAGCAATCGCATTCACACTC
TT8	5′- GCGGAAGTTACGGCTGAAGAG	5′- TGGCATCCCAGAAGGAGGTTC
Tubulin	GGAATGGATACCGAACAACG	CAACGCTAGTCTCAGCAGCA
